# 

**Published:** 1891-10

**Authors:** 


### Ohio State Dental Meeting.

The annual meeting of the Ohio State Dental Society will be held in Columbus, on Tuesday, the first day of December, in the Chittenden Hotel. The members of the Executive Committee are bestirring themselves to make this, one of the best meetings of this Society that has ever been held. It is one of the older State Dental Societies, and should increase in strength and facility of work as it gains experience; this perhaps, has in a sense been done, and after all, the way now seems open for it to accomplish much more than ever before. It is to be hoped that every member will put forth special effort for the interest of this meeting, in securing so far as possible, the attendance of the best men in the profession throughout the State, enlisting their interest, sufficiently at least, to become members; and in addition to this, let everyone go to the meeting prepared to do something to add to its interest, so that the meeting may be interesting and profitable to all who attend. The intention is, for the meeting to occupy three days, and we will venture the suggestion that it would be well to devote a part, if not the whole of the second day, to clinics and demonstrations. A large proportion of those who attend our State Societies, greatly desire to see and learn by actual practical demonstration, and where there is an assurance of a good clinic being given, many will be attracted, who without clinics would not care to be present. In State and District Societies where good clinics are made a feature, there is always found a large attendance. The clinics connected with the Second District Society of New York have become known, and are popular throughout the whole country; men going oftentimes from all parts of the country to witness their clinics. Why shall not the Ohio State Dental Society have as good a clinic as the best? It is to be hoped the Executive Committee will take this matter into serious consideration, and then organize a clinic that shall be a credit to the Society.

The society at its last annual meeting took some steps looking to the organization of a permanent clinical institute. The committee that was appointed has had the matter under advisement. Considering the various details involved in such a work, certain facilities were in prospect that have not yet become available, but which it is hoped in the near future may be so. This consisted in a building or rooms adapted for such a purpose, and for a museum and library as well; the securing of which was a part of the work of the committee.

It is to be hoped at this meeting of the society additional interest will be aroused, and effort put forth for the early establishment of these things; which would in reality be a department of the State Society.

We shall hope very soon to receive the programme for the coming meeting.

### Examiner's Meeting.

The annual meeting of the Board of Dental Examiners will be held at the Chittenden Hotel in Columbus on Tuesday December 1st, 1891 at 12 M.

It is important that any, who may be interested should be promptly present at that time, and not only this, but arrange to remain until the work of the Board is satisfactorily accomplished.

It is to be hoped that the day of the examination of nongraduates as a way of entering the practice of dentistry is rapidly drawing to a close. The facilities are now so great for thorough systematic professional training, that the old superficial method should be abandoned.

### CONTENTS.

### COMMUNICATIONS.

### THE DENTAL REGISTER,

A MONTHLY MAGAZINE DEVOTED TO THE INTERESTS OF THE

### DENTAL PROFESSION.

*Terms Reduced to $2.00 Per Year. Single Copies, 25 Cents.*

### EDITED BY PROF. J. TAFT, D.D.S.

—AND—

### Published by SAM'L A. CROCKER & CO., Ohio Dental and Surgical Depot.

The volume commences in January and closes in December.

The REGISTER being issued monthly is always fresh, therefore Indispensable to the practitioner who desires to keep posted up with the new inventions and improvements which are daily developed. Neither expense nor effort will be spared to give value and variety to its contents. Articles will be illustrated with well executed engravings whenever they will add to the interest or assist to explanation.

Its extensive circulation and frequency of issue make it one of the BEST DENTAL ADVERTISING MEDIUMS in the United States.

All subscriptions must begin with January or July.

Local or special notices, five lines or less, will be inserted for $3 each insertion; over five lines, 50 cts. per line.

All advertising bills payable in advance, or at furthest, after the issue of the first number containing the advertisement. All transient advertisements to be paid for in advance.

☞ CONTRIBUTIONS SOLICITED.

Exclusively Editorial Communications should be addressed to the Editor.

The latest number of the REGISTER may be ordered with or without other goods at any time, and all letters should be addressed to

**SAM'L A. CROCKER & CO., Ohio Dental and Surgical Depot, Cincinnati, O.**

